# Association of HMGCR inhibition with rheumatoid arthritis: a Mendelian randomization and colocalization study

**DOI:** 10.3389/fendo.2023.1272167

**Published:** 2023-11-17

**Authors:** Li Ma, Yufei Du, Chao Ma, Ming Liu

**Affiliations:** ^1^ Department of Endocrinology and Metabolism, Tianjin Medical University General Hospital, Tianjin, China; ^2^ Department of General Practice, Heze Municiple Hospital, Heze, Shandong, China; ^3^ Department of Urology, Heze Municiple Hospital, Heze, Shandong, China

**Keywords:** HMG-CoA reductase, rheumatoid arthritis, statin, Mendelian randomization, genetic colocalization

## Abstract

**Objective:**

The objective of this study was to investigate the association between hydroxymethylglutaryl coenzyme A reductase (HMGCR) inhibition and rheumatoid arthritis (RA) using drug-target Mendelian randomization (MR) and genetic colocalization analyses.

**Methods:**

Two sets of genetic instruments were employed to proxy HMGCR inhibitors: expression quantitative trait loci (eQTLs) of target genes from the eQTLGen Consortium and genetic variants associated with low-density lipoprotein cholesterol (LDL-C) levels with HMGCR locus from open genome-wide association studies (GWAS). Positive control analyses were conducted on type 2 diabetes and coronary heart disease, and multiple sensitivity analyses were performed.

**Results:**

Genetically proxied expression of eQTL was associated with a lower risk of RA (OR=0.996, 95% CI =0.992–0.999, p= 0.032). Similarly, hydroxymethylglutaryl coenzyme A reductase (HMGCR)-mediated low-density lipoprotein cholesterol was negatively associated with risk of RA (OR=0.995, 95% CI =0.991–0.998, p= 0.007) in the inverse variance weighted (IVW) method. Colocalization analysis suggested a 74.6% posterior probability of sharing a causal variant within the SNPs locus (PH4 = 74.6%). A causal relationship also existed between HMGCR-mediated LDL and RA risk factors. The results were also confirmed by multiple sensitivity analyses. The results in positive control were consistent with the previous study.

**Conclusion:**

Our study suggested that HMGCR inhibition was associated with an increased risk of RA while also highlighting an increased risk of current smoking and obesity. These findings contribute to a growing body of evidence regarding the adverse effects of HMGCR inhibition on RA risk, calling for further research on alternative approaches using HMGCR inhibitors in RA management.

## 1 Introduction

Hydroxymethylglutaryl coenzyme A reductase (HMGCR) inhibitors are a widely prescribed class of lipid-lowering drugs. These medications have been shown to significantly reduce mortality by up to 30% in patients diagnosed with coronary artery disease. The current HMGCR inhibitors used in clinical practice belong to the class of statin medications (1, 2). In addition to their lipid-lowering effects, statins possess potent antioxidant and anti-inflammatory properties (3). Experimental studies have suggested that statins may exert influence on immune responses and cellular apoptosis, regulate cytokine production, and modulate endothelial function (4, 5). These effects have sparked interest in understanding the potential role of statins in the management of rheumatoid arthritis (RA).

RA is a chronic autoimmune disease characterized by joint inflammation, pain, and swelling (6). The prevalence of RA has been rising since 1990 (7). It has a considerable impact on both the individual and society (8), including work disability, decline in physical function, and quality of life (9). Despite extensive research, the exact etiology of RA remains elusive.

Observational studies have explored the association between statin use and the risk of developing RA. However, the findings have been inconsistent and controversial. Some studies have suggested a reduced risk of RA associated with long-term statin use or high-intensity statin treatment (10), while others have reported no significant association (11). Conversely, certain studies have hinted at an increased risk of RA among individuals using statins (12). However, limitations such as confounding factors, reverse causality, and measurement biases in observational studies necessitate further investigation using more robust study designs.

Mendelian randomization (MR) analysis represents a powerful method to provide causal evidence for drug-target associations (13). Drug-target MR used genetic instruments mimicking the effect of the drug (14) to explore the pleiotropic outcomes and investigate the potential adverse effects (15). By leveraging genetic variants as instrumental variables, MR analysis can circumvent many of the limitations of observational studies, such as confounding factors and reverse causality, because genes are randomly assigned to the individual at birth (16). In the context of assessing the relationship between statin use and rheumatoid arthritis risk, employing a drug-target MR design could provide crucial insights into the potential causal nature of this association.

In this study, we aimed to investigate the association between HMGCR inhibition and the risk of developing RA using drug-target MR analysis and genetic colocalization techniques. Due to the primary targeting of HMGC reductase by statin medications, we specifically focused on statins. These results contribute to the existing literature on the association between lipid-lowing drug use and RA risk. Such insight is particularly relevant given the widespread use of statins in clinical practice for lipid-lowering purposes.

Our study design allows us to overcome the inherent limitations of traditional observational studies and provide more reliable evidence regarding the potential role of statins in RA risk.

## 2 Methods

### 2.1 Study design

**Figure 1:**
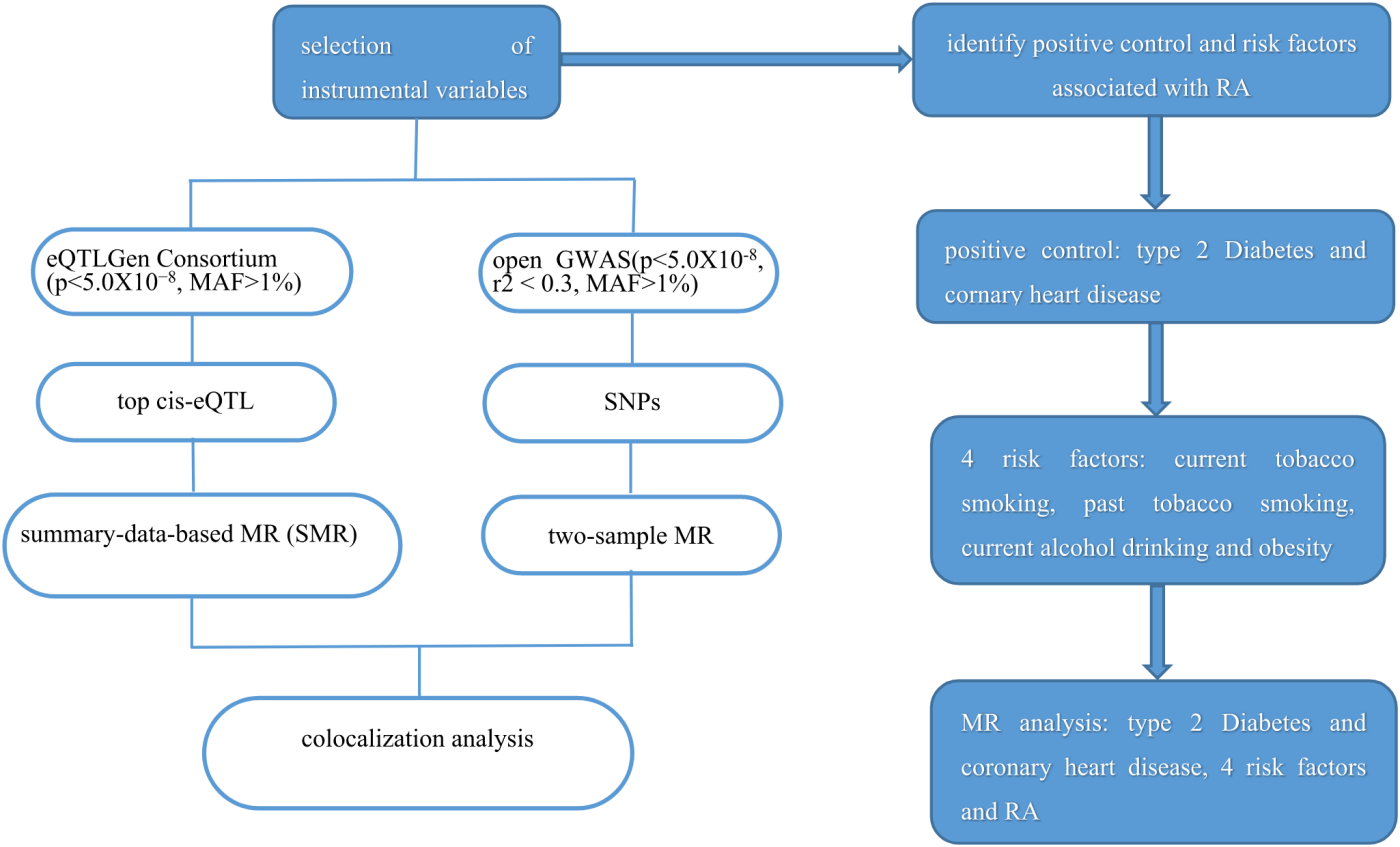
Study flame of the drug target Mendelian randomization and colocalization study. RA, rheumatoid arthritis.

Since the data in our study was based on a public database, no ethics approval was needed. The study frame chart is presented in **Figure 1**.

### 2.2 Selection of genetic instruments

We employed two sets of instrumental variables in our study. First, we obtained expression quantitative trait loci (eQTLs) data for the target genes from the eQTLGen Consortium (https://www.eqtlgen.org/). Among these, only cis-eQTL single-nucleotide polymorphisms (SNPs) that showed significant association with the expression of HMGCR in blood (p < 5.0×10^−8^, minor allele frequency [MAF] > 1%) were selected. Additionally, traditional drug target MR analysis was conducted to validate our study. We identified SNPs located within a 100-kb region surrounding the HMGCR gene that were associated with low-density lipoprotein (LDL) cholesterol levels. This was based on predefined criteria (p < 5×10^−8^, r^2^ < 0.3, MAF > 1%). The summary data of LDL were obtained from the open GWAS, which included 173,082 participants. In summary, seven SNPs were selected for proxying HMGCR inhibitors.

### 2.3 Positive control

To verify the reliability of the selected eQTLs, we performed a positive control study. As statins primarily act to lower LDL cholesterol levels, we utilized LDL cholesterol levels as a positive control in our study. For traditional MR, we used type 2 diabetes and coronary heart disease as the positive control outcomes. The effect of statins on type 2 diabetes and coronary heart disease had already been established in previous studies (17).

### 2.4 GWAS summary data for RA

We obtained the summary data of RA from the Open GWAS database (https://gwas.mrcieu.ac.uk/), which included 462,933 participants (5,201 cases and 457,732 controls). Multiple environmental and lifestyle have been reported to have an association with the development of RA, such as cigarette smoking (18), moderate alcohol intake (19), and obesity (19). Summary data of the RA risk factors were obtained from the Open GWAS database. The data included in our study were based on the European population. Details of all GWASs included in our study are represented in **Table 1**.

**Table 1:** Details of the GWASs included in the drug-target Mendelian randomization.

Phenotype	Detailed information of GWAS data Consortium	Sample size
eQTL of HMGCR	eQTLGen Consortium	31,684 Participants
LDL	Global Lipids Genetics Consortium	173,082 Participants
Type 2 Diabetes	NA	61,714 cases and 1,178 controls
Coronary heart disease	CARDIoGRAMplusC4D	60,801 cases and 123,504 controls
Current smoking	MRC-IEU	424,960 participants
Past smoking	MRC-IEU	32,735 participants
Current alcohol	NA	336,919 cases and 23,807 controls
Obesity class 1	GIANT	32,858 cases and 65,839 controls
Obesity class 2	GIANT	9,899 cases and 62,657 controls
Obesity class 3	GIANT	2,896 cases and 47,468 controls

LDL, low-density lipoprotein cholesterol.

### 2.5 Statistical analysis

Summary-data-based MR (SMR) method was applied to explore the effect of the expression of drug target genes and outcome (20). The HMGCR cis-eQTL was used as an instrument to investigate the association between genetically proxied HMGCR expression and RA. For traditional MR, three different methods of MR (random-effect inverse variance weighted (IVW), MR Egger, and weighted median) were performed to analyze the effect. The IVW was used as the main method. In addition, the association between genetically proxied statins with RA risk factors was evaluated. SNPs included in the traditional MR analysis were selected for colocalization analysis.

Sensitivity analysis was necessary in MR studies. For the SMR analysis, the HEIDI test of p < 0.01 indicates that the association is probably due to linkage (21). For the traditional MR, Cochran Q from the IVW and MR-Egger methods were used to assess potential horizontal pleiotropy. The MR-Egger intercept was an indicator for directional pleiotropy (22). All results were displayed as OR and 95% confidence interval (CI), and p values < 0.05 were considered statistically significant. Analyses were implemented by the package smr-1.3.1, TwoSampleMR (version 4.2.3), and R package Coloc (version 5.2.2).

## 3 Results

### 3.1 The main drug target results

The F statistics of genetic instruments ranged from 50.9 to 372.1 in this study (**Supplementary Table S1**), indicating that our results are unlikely to be affected by weak instruments (23). We selected the most significant cis-eQTL SNP (rs6453133) to proxy for the HMGCR gene. The SMR analysis found a causal relationship between the expression of the HMGCR gene in blood and the risk of RA (OR=0.996, 95% CI =0.992–0.999, p= 0.032). The results indicated that HMGCR inhibitors might increase the risk of RA. For the positive control analysis, the cis-eQTL was associated with LDL cholesterol levels (OR=1.483, 95% CI =0.992–0.999, p= 0.032), indicating the liability of the genetic instrument.

For the traditional drug target MR analysis, the IVW showed a causal relationship between HMGCR-mediated LDL cholesterol and risk of RA (OR=0.995, 95% CI =0.991–0.998, p= 0.007), a similar result was observed in the weighted median method (OR=0.996, 95% CI =0.991–0.999, p= 0.044), further supporting a possible higher risk of HMGCR inhibitors on RA. Positive control analysis demonstrated that LDL cholesterol mediated by gene HMGCR was significantly associated with a high risk of coronary heart disease (OR=1.442, 95% CI =0.992–0.999, p= 2.18e−06). However, it was negatively associated with the risk of type 2 diabetes (OR=0.981, 95% CI =0.973–0.988, p= 1.930e−07), which was consistent with a previous study (24). The results from positive control analysis showed the liability of genetic instruments.

### 3.2 The association between HMGCR-mediated LDL and RA risk factors

**Figure 2:**
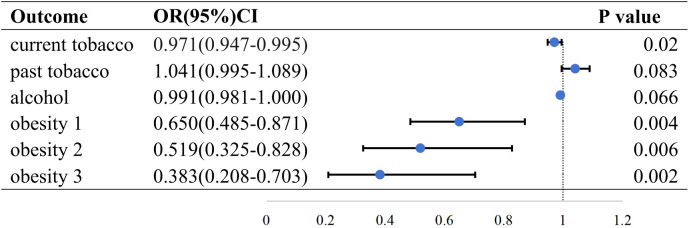
Causal effect of HMGCR mediated LDL on RA risk factors in the IVW method. Forest plots showing the range of OR values for different serological indicators. The vertical lines on either side of the point represent the 95% confidence interval. current: current tobacco smoking; past: past tobacco smoking; alcohol: current alcohol drinking; OR, odds ratio; 95%CI, 95% confidence interval.

Three methods (IVW, MR Egger, and weighted median) were applied to evaluate the association between HMGCR-mediated LDL and several RA risk factors. The results of the IVW method are as follows. Current tobacco smoking (OR=0.971; 95% CI, 0.947 to −0.995; p = 0.020), past tobacco smoking (OR=1.041; 95% CI, 0.995–1.089; p = 0.083), current alcohol drinking (OR=0.991; 95% CI, 0.981–1.000; p = 0.066), obesity class 1 (defined as BMI ≥ 30 kg/m^2^; OR=0.650; 95% CI, 0.485–0.871; p = 0.004), obesity class 2 (defined as BMI ≥ 35 kg/m^2^; OR=0.519; 95% CI, 0.325–0.828; p = 0.006), obesity class 3 (defined as BMI ≥ 40 kg/m^2^; OR=0.383; 95% CI, 0.208–0.703; p = 0.002) (**Figure 2**). The results showed that HMGCR inhibition was associated with an increased incidence of current smoking and obesity.

### 3.3 Colocalization analysis

We conducted SNP-level colocalization analysis to evaluate evidence of a shared causal variant between the HMGCR gene and RA by the coloc. Each configuration yielding posterior probabilities in the colocalization analyses includes five hypotheses: H0, no association with either trait; H1, association with trait 1, not with trait 2; H2, association with trait 2, not with trait 1; H3, association with trait 1 and trait 2, two independent SNPs; and H4, association with trait 1 and trait 2, one shared SNP (25). A posterior probability for H4 (PP.H4) of at least 50% suggests likely to colocalize, and a PP.H4 of at least 80% suggests highly likely to colocalize (26). The 0.5 < PH4 < 0.8 was defined as medium colocalization indication (27). We performed Bayesian colocalization analyses to prioritize SNPs located ± 500 kb from the HMGCR gene and the risk of RA. Colocalization analysis suggested that SNPs associated with LDL and RA had a 74.6% posterior probability of sharing a causal variant within the HMGCR locus (PH1 = 25%, PH2 = 0.00%, PH3 = 0.32%, and PH4 = 74.6%).

### 3.4 Sensitivity analysis

**Figure 3:**
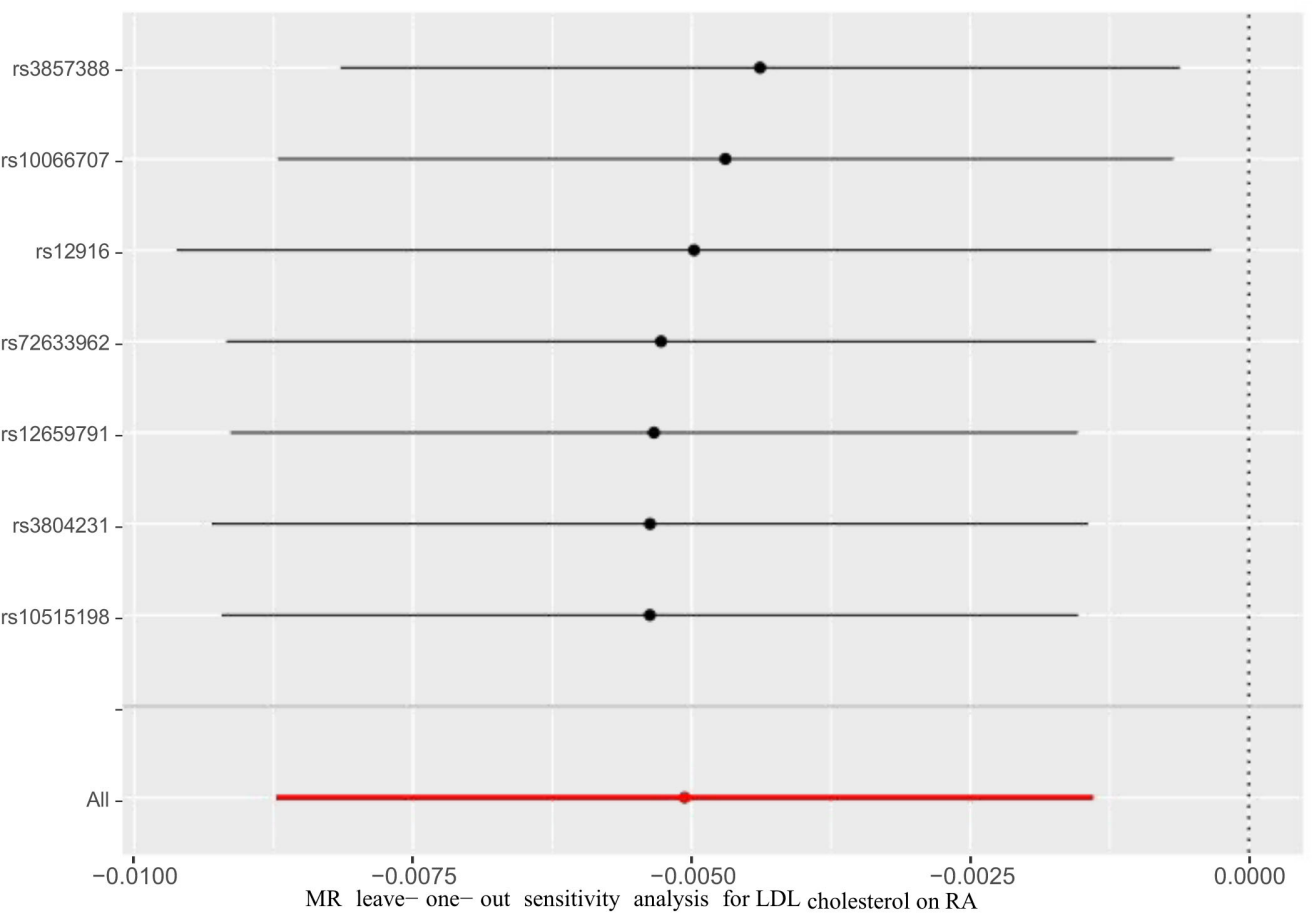
Leave-one-out analysis of the HMGCR mediated LDL cholesterol and RA.

For SMR analysis, the HEIDI test suggested that the association between the expression of HMGCR and the risk of RA was not due to a linkage (p= 0.650). Cochran *Q*-test indicated no evidence of heterogeneity in IVW and MR-Egger methods (p=0.738, p=0.786, respectively). No evidence of pleiotropy was found based on the MR-Egger intercept (p=0.541). Furthermore, a leave-one-out sensitivity test was performed, indicating that the result was not biased by a single SNP (**Figure 3**).

## 4 Discussion

In this study, we conducted a drug-target Mendelian randomization (MR) analysis to assess the association between HMGCR inhibition and the risk of rheumatoid arthritis (RA). By leveraging genetic instruments, we aimed to overcome the limitations of observational studies and provide more reliable evidence regarding the potential effects of HMGCR inhibitors on RA development. Our findings suggest a potential link between HMGCR inhibition and the risk of RA. The MR analysis using genetically proxied HMGCR inhibitors exposure showed a statistically increased risk of developing RA. The results were consistent in three methods, indicating the accuracy of our study. We also performed different sensitivity analyses to confirm the reliability of our results. Multiple sensitivity analyses showed no pleiotropy and heterogeneity. Leave-one-out sensitivity test was also applied, and the result demonstrated that the causal association might not be biased by a single SNP. Additionally, colocalization analysis provided further support for the presence of a shared causal variant within the loci associated with HMGCR inhibitors and RA, strengthening the plausibility of a causal relationship.

Several studies investigating lipid metabolism in RA patients have yielded inconsistent results. A retrospective study reported lower levels of total cholesterol (TC) and LDL cholesterol before the RA incidence (28). In contrast, some studies have found increased total cholesterol levels in RA patients (29). Lipid metabolism with the treatment of disease-modifying antirheumatic drugs (DMARDs) and biological therapies was complex. Biological therapies demonstrated an atheroprotective effect via maintaining the level of reverse cholesterol transport (RCT) proteins or anti-atherogenic lipid apoA1 (30, 31), while different anti-rheumatic disease medications have varying effects on lipid metabolism. Studies have indicated that biological disease-modifying drugs have a protective effect against atherosclerosis, while corticosteroids have a proatherogenic effect (32). Karpouzas et al. propose that the use of statin medications is associated with a long-term reduction in cardiovascular risk in patients with RA who have higher levels of inflammation. However, there is currently a lack of research on the risk of developing RA in relation to statin use (33).

The mechanisms underlying the observed association between HMGCR inhibition and RA risk remain unclear. Studies have suggested that statins possess immunomodulatory properties and may impact immune responses, leading to dysregulation and potentially triggering autoimmune processes (34, 35). In addition, statins strongly sensitized the cells to apoptotic agents (36). Therefore, statin use may play a role in the development of RA through the chain of immunogenic reactions (37, 38). Among environmental factors, smoking has been the strongest risk factor for the development of rheumatoid arthritis (39). Our research findings demonstrate an association between HMGCR inhibitors and an increased prevalence of current smoking, suggesting that they may potentially influence the risk of developing RA through affecting current smoking or other related factors. However, further investigation is required to elucidate the precise molecular mechanisms involved in this association.

It is noteworthy that patients with RA are at an increased risk of cardiovascular events compared to the general population (40), and cardiovascular (CV) events are the leading cause of death in patients with RA(10%–30% of deaths) (41). Statins are commonly prescribed to manage cardiovascular risk factors in these individuals. Our study identified an increased risk of RA associated with statin use. It is important to consider the well-established cardiovascular benefits of statins. The decision to initiate or continue statin therapy should be carefully weighed, taking into account both the potential risks and the cardiovascular protective effects.

Our study has several strengths, including the utilization of two sets of genetic instruments to proxy drug exposure, the incorporation of colocalization analysis, the inclusion of positive control analyses, and the performance of multiple sensitivity analyses. These measures ensure the robustness and validity of our results.

However, some limitations should also be acknowledged. Generalizability may be limited as our study included predominantly individuals of European ancestry. Additionally, it is true that drug-target MR studies may not fully capture the real effects of drugs, as they cannot account for confounding factors such as drug dose and metabolism, mechanism of action, individual differences, and duration of drug exposure. Thus, the activity of HMGCR may not fully represent the complete range of effects caused by statin medications. While HMGCR activity is a significant factor in the mechanism of action for statins, it does not capture all the complexities and nuances associated with statin therapy, such as potential off-target effects, individual variations in response, and diverse molecular pathways affected by statins. Therefore, to understand the comprehensive effects of statins, it is essential to consider multiple factors beyond just HMGCR activity (21). Further research, including clinical trials and experimental studies, is necessary to address these limitations and provide a more comprehensive understanding of the potential adverse effects of HMGCR inhibition on RA risk.

In conclusion, our drug-target MR analysis suggests an increased risk of developing rheumatoid arthritis associated with HMGCR inhibition, the target enzyme of statins. Although the results cannot reflect the real effects of drugs, they may hint at evidence in evaluating the long-term effects of drugs. Our study provides robust evidence of a potential adverse effect of HMGCR inhibition on RA susceptibility. These findings have important implications for the safe use of statins, particularly in individuals at high risk of RA.

Further investigation is warranted to elucidate the underlying mechanisms and determine the clinical implications of these findings. The evidence presented here calls for a cautious approach towards the re-purposing or re-targeting of HMGCR inhibitors and highlights the need for careful consideration of personalized treatment strategies in individuals at risk of both RA and cardiovascular diseases.

## Data availability statement

The original contributions presented in the study are included in the article/**Supplementary Material**. Further inquiries can be directed to the corresponding author.

## Ethics statement

Ethical approval was not required for the study involving humans in accordance with the local legislation and institutional requirements. Written informed consent to participate in this study was not required from the participants or the participants’ legal guardians/next of kin in accordance with the national legislation and the institutional requirements.

## Author contributions

ML: Writing – review & editing. LM: Writing – original draft. YD: Writing – original draft. CM: Writing – original draft.
