# Supplementary material for: Association of HMGCR inhibition with rheumatoid arthritis: a Mendelian randomization and colocalization study
Source: Front Endocrinol (Lausanne). 2023 Nov 17;14:1272167. doi: 10.3389/fendo.2023.1272167 (PMC10691537; doi:10.3389/fendo.2023.1272167)
Supplement: Supplementary file 1 [file Table_1.docx]

| **SNP** | **Chr** | **EA** | **OA** | **EAF** | **beta** | **se** | **p value** | **F-statistic** |
| --- | --- | --- | --- | --- | --- | --- | --- | --- |
| rs10066707 | 5 | A | G | 0.4169 | 0.0497 | 0.0054 | 2.97E-19 | 84.7 |
| rs3804231 | 5 | A | G | 0.1319 | 0.0642 | 0.0053 | 1.88E-29 | 146.7 |
| rs10515198 | 5 | A | G | 0.1029 | 0.0599 | 0.0061 | 5.99E-22 | 96.4 |
| rs12659791 | 5 | C | T | 0.1557 | 0.0433 | 0.005 | 1.42E-18 | 75.0 |
| rs12916 | 5 | C | T | 0.4314 | 0.0733 | 0.0038 | 7.79E-78 | 372.1 |
| rs72633962 | 5 | C | T | 0.1412 | 0.06 | 0.0072 | 3.33E-15 | 69.4 |
| rs385738 8 | 5 | C | T | 0.1280 | 0.0421 | 0.0059 | 2.20E-11 | 50.9 |

**Supplementary table 1.** Information of genetic instrumental variants associated with LDL cholesterol located within 100 kb windows from gene HMGCR.

SNP, single-nucleotide polymorphism; Chr, Chromosome; EA, Effect allele; OA, Other allele; EAF, Effect allele frequency; SE, Standard error;
